# Dual‐Phase Efficacy of a Repair Cream Containing Dexpanthenol, Recombinant Human Fibronectin Peptide, and *Coreopsis tinctoria* Extract Following 1565‐Nm Non‐Ablative Fractional Laser in Sensitive Skin: A Randomized Split‐Face Trial

**DOI:** 10.1111/jocd.71220

**Published:** 2026-10-01

**Authors:** Qiaoyuan Liu, Xueni Lin, Meixin Lu, Peng Shu, Meiting Li, Zhenan Yao, Rong Hu

**Affiliations:** ^1^ HBN Research Institute and Biological Laboratory Shenzhen Hujia Technology Co. Ltd. Shenzhen Guangdong China; ^2^ Guangdong Engineering Technology Research Center for Functional Skincare Innovation Shenzhen Hujia Technology Co. Ltd. Shenzhen Guangdong China

**Keywords:** coreopsis tinctoria, dexpanthenol, high‐frequency ultrasound, nonablative fractional laser, recombinant human fibronectin peptide, sensitive skin, skin barrier repair

## Abstract

**Background:**

The 1565‐nm non‐ablative fractional laser (NAFL) induces dermal thermal damage while preserving epidermal integrity, producing immediate postoperative erythema, edema, and barrier disruption. The ultra‐early 0–2‐h recovery window remains poorly characterized by quantitative data, and medium‐term post‐laser trials lack objective dermal endpoints.

**Objective:**

To evaluate the dual‐phase efficacy and safety of a repair cream containing dexpanthenol, recombinant human fibronectin peptide (rhFNP), and *Coreopsis tinctoria* extract following 1565‐nm NAFL treatment.

**Methods:**

A single‐center, randomized, investigator‐blind, split‐face trial enrolled 33 females with sensitive and barrier‐impaired skin. Participants received full‐face 1565‐nm NAFL. For short‐term assessment, one side received the repair cream while the contralateral side remained untreated. For medium‐term evaluation, sides were randomized to the repair cream or the standard‐care comparator. Endpoints included transepidermal water loss (TEWL), hydration, erythema index (EI), blood perfusion, surface temperature, and dermal thickness/density.

**Results:**

Within 2 h, the repair cream reduced TEWL by 4.63% at 30 s and 3.94% at 2 h, increased hydration by 89.76% at 30 s, lowered EI by 5.96%, perfusion by 17.85%, and surface temperature by 2.15%, all at 10 min (each *p* < 0.001). Over 14 days, TEWL decreased by 4.34% versus 1.12% on the comparator side, with skin thickness increasing by 12.67% versus 6.62% and density by 17.70% versus 0.15%. No adverse events occurred.

**Conclusion:**

This repair cream mitigates acute inflammatory responses within 2 h and is associated with favorable early changes in ultrasonographically assessed dermal parameters over 14 days, offering a post‐NAFL care option for sensitive skin populations.

## 1 Introduction

Non‐ablative fractional lasers (NAFLs), particularly the 1565‐nm ResurFX, have become a mainstay in aesthetic dermatology for skin rejuvenation. The 1565‐nm wavelength induces controlled thermal injury deep within the dermal layer, promoting neocollagenesis and progressive collagen remodeling. This mechanism underlies its widespread clinical applications, including facial rejuvenation, rhytide improvement, correction of atrophic post‐acne scars, and management of superficial scars [1, 2, 3, 4, 5, 6]. Despite a favorable safety profile, the procedure consistently elicits immediate postoperative reactions. Nearly all patients experience immediate post‐treatment erythema and edema, which typically last from 2 to 4 days, accompanied by sensations of burning or stinging and mild‐to‐moderate pain [7, 8, 9, 10, 11]. These cutaneous changes result directly from photothermal injury, which triggers oxidative stress, the release of pro‐inflammatory cytokines, and transient disruption of the skin barrier [12, 13, 14]. If not managed appropriately, this physiological cascade can extend recovery time and elevate the risk of complications.

Importantly, the clinical demand for NAFL extends well beyond individuals with robust skin. Self‐reported sensitive skin affects approximately 50% of Chinese women [15], and this population seeks the same aesthetic improvements, including pore refinement, scar attenuation, and photorejuvenation, that NAFL is established to address. Compared with ablative fractional lasers that create full‐thickness epidermal wounds, NAFL preserves epidermal integrity [16] and thus represents a comparatively favorable procedural option for barrier‐vulnerable individuals. Nevertheless, their characteristically fragile barrier and heightened neurovascular reactivity [17] predispose sensitive‐skin patients to more intense post‐procedural erythema, edema, and elevated transepidermal water loss relative to non‐sensitive counterparts. Whether NAFL can be safely delivered in this population, and which post‐procedural care strategies optimize barrier recovery, remains an important practical clinical question that the present study directly addresses.

Answering this question required closing two distinct evidence gaps in the current literature, each corresponding to a specific phase of post‐NAFL recovery. The first concerns the ultra‐early window (0–2 h). This interval may represent a critical period during which photothermal injury triggers rapid inflammatory amplification that, if left unmodulated, extends recovery time and elevates complication risk [13, 14]. Although a limited number of studies using related non‐ablative platforms have captured erythema at discrete early time points [18], no trial has performed serial dynamic biophysical monitoring across the complete 0–2‐h interval following 1565‐nm NAFL, and the majority of efficacy evaluations commence only at 24 h or later [6, 7, 19]. The second gap concerns the medium‐term phase (14 days). Existing post‐laser skincare trials have predominantly employed superficial epidermal indicators, including transepidermal water loss (TEWL), stratum corneum hydration, and erythema index (EI), as efficacy endpoints [6, 18, 20, 21, 22]. Because NAFL exerts its therapeutic effect within the dermis, evaluating an intervention solely through these epidermal surrogates provides no direct measure of its impact on the laser‐treated compartment. Dermal structural endpoints are therefore required, yet objective quantification of these parameters has remained largely absent from this literature. Both gaps are compounded in sensitive‐skin populations, who remain under‐represented in controlled post‐procedural research despite their heightened reactivity and fragile barrier [17]. To our knowledge, no randomized split‐face trial has evaluated an early repair strategy across both temporal phases in this demographic. The present study was designed to bridge these dual gaps through serial 0–2‐h monitoring and 14‐day combined epidermal and ultrasonographic dermal assessment, employing a bioactive repair cream whose mechanisms address the divergent physiological demands of both phases.

Bioactive‐enriched topical creams represent a clinically feasible strategy to address the multifaceted ultra‐early repair needs following 1565‐nm NAFL treatment. Dexpanthenol (provitamin B5) demonstrates effective percutaneous penetration, accelerates re‐epithelialization, enhances stratum corneum hydration, reduces TEWL, and exerts anti‐inflammatory effects that facilitate acute wound repair [20, 23, 24, 25, 26]. Recombinant human fibronectin peptide (rhFNP) promotes the migration of keratinocytes and fibroblasts, stimulates extracellular matrix (ECM) synthesis, reinforces epidermal barrier integrity, and downregulates pro‐inflammatory cytokine expression [27, 28]. *Coreopsis tinctoria* Nutt. is abundant in flavonoids with potent antioxidant and anti‐inflammatory properties [29, 30, 31]. Its major active constituent, okanin, alleviates intracellular oxidative damage, inhibits pro‐inflammatory cytokine secretion, and enhances collagen synthesis, thereby mitigating photodamage and accelerating skin repair [32, 33]. These three ingredients operate through complementary pathways: dexpanthenol emphasizes acute skin barrier reconstruction, rhFNP supports cellular migration and dermal matrix deposition, and *Coreopsis tinctoria* extract alleviates oxidative stress and inflammation. Collectively, they address all key physiological changes during the post‐laser golden window.

In this context, we hypothesize that the immediate topical application of this triple‐ingredient compound repair cream following 1565‐nm NAFL treatment can effectively mitigate acute thermal stress and subsequent inflammatory responses within the 0–2‐h window in patients with sensitive skin. Continued use of the product is anticipated to promote earlier normalization of ultrasonographically assessed dermal parameters relative to the comparator regimen over the first 14 days. Findings from this split‐face controlled trial will provide quantitative clinical evidence to guide standardized early postoperative skincare for patients with sensitive skin undergoing NAFL treatment.

## 2 Materials and Methods

### 2.1 Study Design and Ethics

This was a single‐center, randomized, investigator‐blind, intra‐individual split‐face clinical trial conducted between June 2025 and July 2025. Each participant completed a 14‐day study with 8 evaluation time points: baseline (D0), immediately after a single 1565‐nm NAFL treatment before product application (D0′), and then at 30 s (D0T30s), 10 min (D0T10min), and 2 h (D0T2h) after product application, followed by assessments on Day 3 (D3), Day 7 (D7), and Day 14 (D14) post‐treatment. For short‐term ultra‐early efficacy, one side of the face received the test product and the contralateral side remained untreated (open‐label). For medium‐term efficacy, sides were randomly assigned to receive either the test product, or a standard‐care comparator comprising a sodium hyaluronate dressing plus a vehicle‐matched placebo. All outcome assessments were performed by independent, blind investigators unaware of treatment allocation. The study procedure is provided in Figure 1.

**FIGURE 1:**
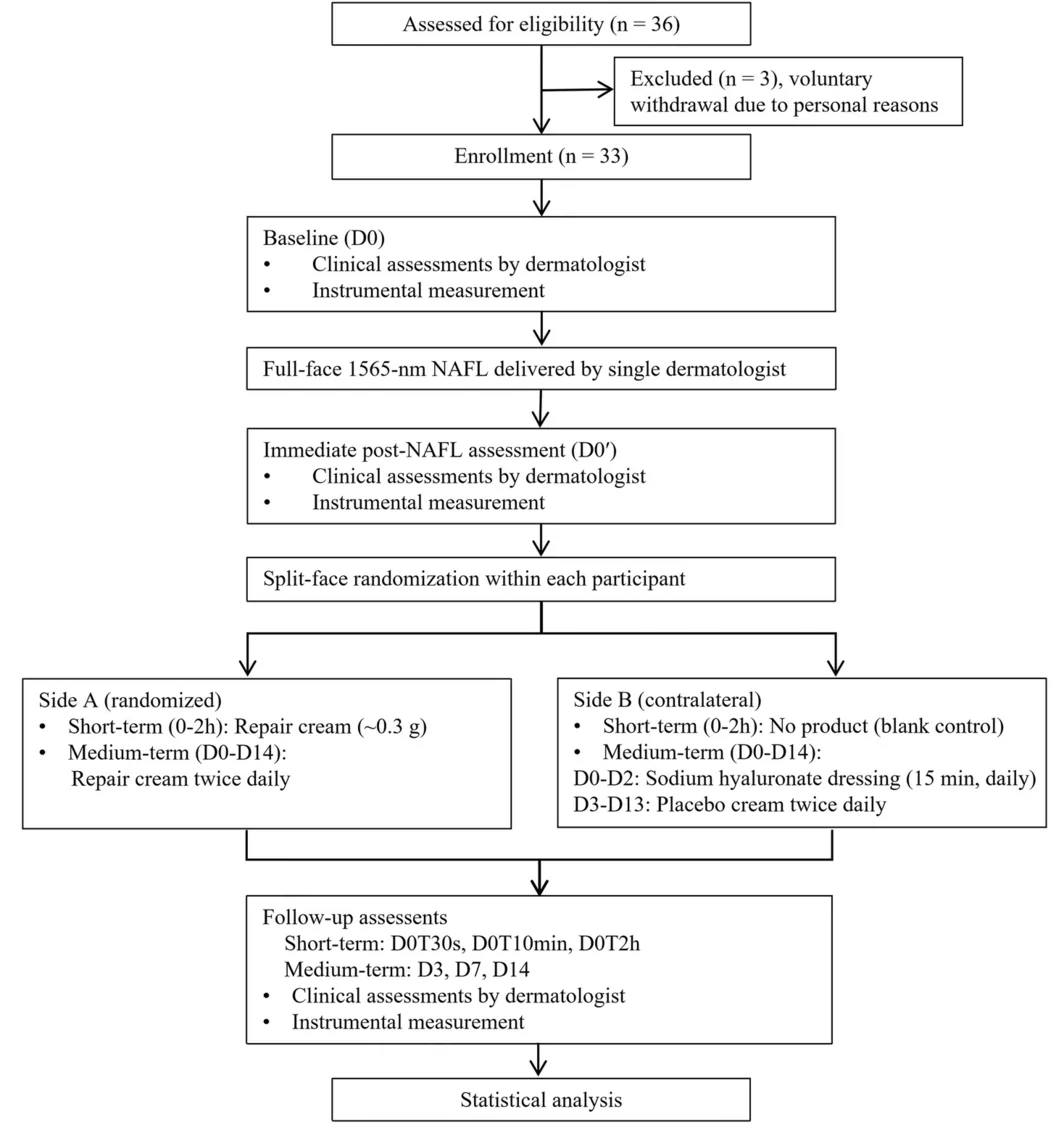
Flowchart of the clinical trial.

This trial was approved by the Ethics Committee of the Medical Laboratory. All procedures adhered to the Declaration of Helsinki and the Measures for Ethical Review of Biomedical Research Involving Humans. Before participating in any study‐related procedures, all participants provided written informed consent following full disclosure of the study objectives, methodologies, potential risks, and their right to withdraw at any time. Participants additionally provided authorization for the publication of study‐related images.

### 2.2 Participants

A total of 36 healthy Chinese females aged 25–50 years were assessed for eligibility. Three withdrew for personal reasons before study commencement. The remaining 33 participants (mean age 37.91 ± 6.95 years) were enrolled and completed the 14‐day study. Inclusion criteria were as follows: healthy Chinese females aged 25–50 years who self‐reported sensitive facial skin, routine sunscreen use, and comparable bilateral facial erythema (grade ≥ 3 per standardized photographic atlas) and flushing, with cheek dryness (Corneometer < 45 c.u.) and TEWL > 15 g/m^2^·h on both cheeks. The treatment area had to be free of visible lesions, scars, or excessive hair.

Participants were excluded if they: (1) were unable to comprehend the informed consent document, refused to provide written informed consent, or were unwilling to adhere to protocol requirements; (2) were concurrently enrolled in another clinical trial; (3) were pregnant, breastfeeding, or planning pregnancy; (4) had infectious dermatoses, atopic dermatitis, or a history of severe reactions or allergies to cosmetics, medications, or light exposure; (5) had nevi, telangiectasia, verrucae, scars, or other cutaneous abnormalities in the treatment area, or had lesions that would interfere with measurements; (6) had applied cosmetics, skincare products, or topical medications in the treatment area on the evaluation day, or had applied functional cosmetics (e.g., soothing or repairing products) in the treatment area within 2 weeks; (7) had changed skincare or cosmetic products in the treatment area within 2 weeks; (8) had undergone minimally invasive procedures (e.g., injectable fillers, botulinum toxin, laser, deep peeling) in the treatment area within 6 months; (9) had received skin peeling, dermatological treatments, immunosuppressive therapy, or used topical or systemic retinoids in the treatment area within 3 months; (10) had received systemic corticosteroids or phototherapy within 1 month; (11) had experienced intense sun exposure or excessive ultraviolet radiation (e.g., mountain climbing, phototherapy, tanning salon use) within 1 week; (12) were deemed unsuitable by the investigator for any other reason.

### 2.3 Grouping and Interventions

At baseline (D0), all participants received full‐face 1565‐nm NAFL treatment using the M22 ResurFX system (Lumenis Ltd., Yokneam, Israel). A topical 5% lidocaine cream was applied to the entire face at a thickness of approximately 2 mm and covered with a transparent occlusive film for 40 min before laser irradiation. After removing the anesthetic cream, the face was cleansed with water and disinfected using standard skin antisepsis. Laser treatment was performed by a single experienced dermatologist with the following standardized parameters: ResurFX mode, 1565‐nm wavelength, energy 35–40 mJ per microbeam, and density 200–250 spots/cm^2^. The ResurFX handpiece was held perpendicular to the skin, and uniform, non‐overlapping, parallel scanning was performed from the lower to upper face until full‐face treatment was completed.

Immediately after NAFL treatment, participants were randomized to receive the repair cream containing dexpanthenol, rhFNP, and *Coreopsis tinctoria* extract on one side (~0.3 g) while the contralateral side received no product (blank control), under technician supervision. For the 14‐day home‐care phase, the repair cream was applied to the assigned side twice daily (morning and evening) after routine cleansing, with gentle massage until fully absorbed. Participants were instructed to use an amount consistent with their habitual skincare practice, which in practice corresponded to approximately 0.3 g per half‐face application. The contralateral side received standard care: a commercially available sodium hyaluronate dressing for 15 min once daily on D0–D2, followed by a vehicle‐matched placebo twice daily from D3 to D13. On follow‐up visit days (D3, D7, and D14), participants were instructed to cleanse with their habitual facial cleanser only and to refrain from applying any study products or sunscreen until all scheduled assessments were completed.

All participants were instructed to apply the provided sunscreen uniformly over the entire face every day commencing on D1 (the second day) after 1565‐nm NAFL treatment, even in the absence of outdoor activity, and to avoid intense sun exposure or travel. For outdoor activities, physical photoprotection (wide‐brimmed hat, umbrella, sun‐protective clothing, and sunglasses) was mandated. Throughout the study, only habitual facial cleansers, the assigned study products, and sunscreen were permitted; no other functional skincare products were allowed.

### 2.4 Endpoints and Assessments

All outcomes were evaluated across two predefined testing phases: an ultra‐early short‐term phase within 2 h after single‐product application and a medium‐term phase following 14 consecutive days of daily administration.

The primary endpoints included TEWL, skin hydration, and EI. TEWL was measured using an open‐chamber Tewameter TM Hex (Courage + Khazaka electronic GmbH, Köln, Germany) to characterize cutaneous barrier function. Skin hydration was assessed with a Corneometer CM825 (Courage + Khazaka) using capacitance methodology. EI was determined with a Mexameter MX18 (Courage + Khazaka) to objectively quantify erythema severity.

The secondary endpoints comprised skin color a* value, erythema area ratio, skin blood perfusion, and skin surface temperature, as well as skin thickness and density. Standardized high‐resolution facial imaging was captured with the VISIA‐CR system (Canfield Scientific, New Jersey, USA) for subsequent quantitative analysis of skin color a* value and erythema area ratio. Real‐time skin blood perfusion was monitored using the PeriCam PSI (Perimed AB, Stockholm, Sweden). Skin surface temperature was measured using an Optris P1400 (Optris GmbH, Berlin, Germany). Skin thickness and density were assessed using the DermaLab Combo 20‐MHz Ultrasound probe (Cortex Technology, Aalborg, Denmark). All outcome parameters and corresponding assessment time points are summarized in Table 1.

**TABLE 1:** Schedule of outcome assessments across the study period.

Parameters	D0	D0′	Short‐term efficacy			Medium‐term efficacy		
			D0T30s	D0T10min	D0T2h	D3	D7	D14
Transepidermal water loss (TEWL) (g/m^2^·h)	✔	✔	✔	✔	✔	✔	✔	✔
Skin hydration (c.u.)	✔	✔	✔	✔	✔	✔	✔	✔
Erythema index (EI) (a.u.)	✔	✔		✔	✔	✔	✔	✔
Skin color a* value (a.u.)	✔	✔	✔	✔	✔	✔	✔	✔
Erythema area ratio (%)	✔	✔	✔	✔	✔	✔	✔	✔
Skin blood perfusion (PU)	✔	✔		✔	✔	✔	✔	✔
Skin surface temperature (°C)		✔		✔	✔			
Skin thickness (μm)	✔	✔					✔	✔
Skin density (a.u.)	✔	✔					✔	✔

*Note:* The symbol ✔ indicates that the parameter was assessed at the corresponding time point. Skin surface temperature was not acquired at D0, because this parameter was designed to investigate short‐term skin temperature changes induced by different interventions immediately following non‐ablative fractional laser (NAFL) treatment.

Abbreviations: a.u., arbitrary units; c.u., Corneometer units; D0, baseline before NAFL treatment; D0′, immediately after NAFL treatment before product application; D0T30s, D0T10min, and D0T2h, 30 s, 10 min, and 2 h after product application; D3, D7, and D14, days 3, 7, and 14 post‐treatment; PU, perfusion units.

### 2.5 Safety Assessment

Participants were continuously monitored for local cutaneous adverse reactions throughout the trial. A dermatologist conducted visual assessments at each visit, and participants were required to report any abnormal skin symptoms. All adverse events were recorded systematically per standard clinical safety procedures for cosmetic research.

### 2.6 Statistical Analysis

All statistical analyses were conducted using IBM SPSS Statistics Version 27.0 (IBM Corp., New York, USA). Normality of data distribution was assessed via the Shapiro–Wilk test. For normally distributed continuous data, paired sample *t*‐tests were employed for within‐side comparisons (versus D0 or D0′) and between‐side comparisons. For non‐normally distributed continuous data, the Wilcoxon signed‐rank test was used for within‐side comparisons and the Mann–Whitney U test for between‐side comparisons. Continuous variables are presented as mean ± standard deviation (SD). Changes from D0 or D0′ are reported as mean difference with 95% confidence intervals (CI). Bonferroni correction was applied to adjust for multiple comparisons across time points. All statistical tests were two‐sided, and a significance level of *p* < 0.05 was predetermined.

## 3 Results

### 3.1 Baseline Characteristics and Immediate Post‐NAFL Biophysical Changes

At baseline (D0), no significant differences were observed between the repair cream and blank control/standard‐care comparator sides for any primary or secondary endpoint (all *p* > 0.05), confirming successful randomization and side comparability (Table 2).

**TABLE 2:** Skin biophysical parameters at baseline (D0) and immediately after NAFL treatment (D0′) on the repair cream and blank control/standard‐care comparator sides.

Parameters	Time point	Mean ± SD		Change from D0		Between‐side *p*
		Repair cream	Blank control/standard‐care comparator	Repair cream	Blank control/standard‐care comparator	
Transepidermal water loss (TEWL) (g/m^2^·h)	D0	17.96 ± 1.40	17.92 ± 1.35	—	—	0.453
	D0′	27.19 ± 2.68	27.32 ± 2.63	9.23 ± 2.02***	9.40 ± 1.90***	0.172
Skin hydration (c.u.)	D0	38.43 ± 3.45	38.79 ± 3.69	—	—	0.112
	D0′	30.09 ± 3.16	30.11 ± 3.17	−8.35 ± 2.88***	−8.68 ± 3.04***	0.908
Erythema index (EI) (a.u.)	D0	331.38 ± 43.90	331.26 ± 43.61	—	—	0.800
	D0′	378.06 ± 43.58	377.74 ± 43.70	46.68 ± 21.51***	46.48 ± 20.16***	0.674
Skin color a* value (a.u.)	D0	22.63 ± 4.48	23.01 ± 5.33	—	—	0.364
	D0′	38.42 ± 7.63	39.70 ± 7.04	15.79 ± 5.17***	16.69 ± 5.22***	0.001^††^
Erythema area ratio (%)	D0	40.21 ± 3.87	39.18 ± 4.61	—	—	0.135
	D0′	43.30 ± 6.43	43.12 ± 5.86	3.06 ± 6.07***	4.00 ± 5.58***	0.748
Skin blood perfusion (PU)	D0	135.47 ± 31.29	136.10 ± 33.57	—	—	0.606
	D0′	339.16 ± 59.91	342.23 ± 57.88	203.69 ± 70.47***	206.13 ± 68.44***	0.203
Skin thickness (μm)	D0	1285.15 ± 185.88	1270.06 ± 174.76	—	—	0.301
	D0′	1645.36 ± 206.38	1631.18 ± 207.87	360.21 ± 151.81***	361.12 ± 137.20***	0.410
Skin density (a.u.)	D0	26.33 ± 6.93	26.48 ± 7.45	—	—	0.686
	D0′	22.53 ± 5.54	22.39 ± 5.37	−3.80 ± 6.38***	−4.08 ± 5.99***	0.758
Skin surface temperature (°C)	D0′	35.86 ± 0.46	35.85 ± 0.44	—	—	0.609

*Note:* Statistical significance of within‐side changes versus D0: ***p < 0.001. Between‐side *p*‐values compare repair cream versus blank control/standard‐care comparator sides: ^††^ *p* < 0.01. Skin surface temperature was not acquired at D0, because this parameter was designed to investigate short‐term skin temperature changes induced by different interventions immediately following NAFL treatment.

Abbreviations: a.u., arbitrary units; c.u., Corneometer units; D0, baseline before NAFL treatment; D0′, immediately after NAFL treatment before product application; NAFL, non‐ablative fractional laser; PU, perfusion units; SD, standard deviation.

Immediately after single‐session 1565‐nm NAFL treatment (D0′), both sides exhibited significant alterations in all measured parameters compared to baseline. TEWL, EI, skin color a* value, erythema area ratio, skin thickness, and skin blood perfusion increased significantly (all *p* < 0.001), whereas skin hydration and density decreased significantly (all *p* < 0.001). Except for the skin color a* value, which was higher on the blank control/standard‐care comparator side than on the repair cream side (39.70 ± 7.04 vs. 38.42 ± 7.63, *p* = 0.001), no significant between‐side differences were detected for any other parameter at D0′ (Table 2).

### 3.2 Short‐Term Effects of Topical Intervention (D0T30s to D0T2h)

Immediately after product application, assessments of skin barrier function, erythema, cutaneous microcirculation, and skin surface temperature were performed at D0T30s, D0T10min, and D0T2h (Table 3).

**TABLE 3:** Short‐term changes of skin biophysical parameters relative to D0′: Repair cream versus blank control split‐face comparison.

Parameters	Time point	Mean ± SD		Change from D0′ [mean difference; 95% CI]		Between‐side *p*
		Repair cream	Blank control	Repair cream	Blank control	
Transepidermal water loss (TEWL) (g/m^2^·h)	D0T30s	25.93 ± 2.80	27.62 ± 2.59	−1.26 (−1.67, −0.85)***	0.30 (0.15, 0.45)**	< 0.001^†††^
	D0T10min	25.57 ± 2.70	27.87 ± 2.53	−1.62 (−1.90, −1.35)***	0.55 (0.38, 0.71)***	< 0.001^†††^
	D0T2h	26.12 ± 2.68	28.13 ± 2.50	−1.07 (−1.36, −0.78)***	0.81 (0.61, 1.01)***	< 0.001^†††^
Skin hydration (c.u.)	D0T30s	57.10 ± 4.73	29.45 ± 3.25	27.01 (25.07, 28.95)***	−0.66 (−0.99, −0.33)**	< 0.001^†††^
	D0T10min	59.87 ± 4.87	29.30 ± 3.11	29.78 (27.75, 31.82)***	−0.80 (−1.19, −0.41)***	< 0.001^†††^
	D0T2h	57.73 ± 4.50	29.14 ± 3.11	27.64 (25.73, 29.56)***	−0.96 (−1.54, −0.39)**	< 0.001^†††^
Erythema index (EI) (a.u.)	D0T10min	355.51 ± 43.77	380.94 ± 44.54	−22.54 (−25.55, −19.54)***	3.20 (2.34, 4.06)***	< 0.001^†††^
	D0T2h	358.05 ± 44.04	383.28 ± 43.69	−20.01 (−23.11, −16.91)***	5.55 (4.31, 6.78)***	< 0.001^†††^
Skin color a* value (a.u.)	D0T30s	37.97 ± 7.99	39.79 ± 7.10	−0.45 (−0.99, 0.09)	0.09 (−0.78, 0.96)	0.643
	D0T10min	37.11 ± 8.29	39.45 ± 7.43	−1.31 (−1.99, −0.64)**	−0.25 (−1.31, 0.82)	0.103
	D0T2h	35.92 ± 8.30	40.00 ± 6.59	−2.50 (−3.19, −1.82)***	0.29 (−0.72, 1.30)	< 0.001^†††^
Erythema area ratio (%)	D0T30s	41.76 ± 4.32	42.52 ± 5.83	−1.58 (−3.13, −0.02)	−0.48 (−1.34, 0.38)	0.488
	D0T10min	41.00 ± 4.02	42.52 ± 5.51	−2.21 (−3.77, −0.65)*	−0.58 (−1.59, 0.44)	0.123
	D0T2h	41.18 ± 3.92	42.36 ± 5.53	−2.12 (−3.98, −0.26)	−0.70 (−1.82, 0.43)	0.387
Skin blood perfusion (PU)	D0T10min	278.60 ± 48.07	327.17 ± 65.20	−60.55 (−73.71, −47.40)***	−15.06 (−28.37, −1.76)	< 0.001^†††^
	D0T2h	306.21 ± 54.36	325.65 ± 58.49	−32.95 (−53.90, −12.00)**	−16.59 (−37.33, 4.16)	< 0.001^†††^
Skin surface temperature (°C)	D0T10min	35.10 ± 0.60	35.64 ± 0.49	−0.77 (−0.96, −0.57)***	−0.21 (−0.34, −0.08)**	< 0.001^†††^
	D0T2h	35.29 ± 0.68	35.52 ± 0.60	−0.57 (−0.76, −0.38)***	−0.33 (−0.49, −0.18)***	< 0.001^†††^

*Note:* Statistical significance of within‐side changes versus D0′: *p < 0.05, **p < 0.01, ***p < 0.001. Between‐side *p*‐values compare repair cream versus blank control sides: ^†††^ *p* < 0.001.

Abbreviations: a.u., arbitrary units; c.u., Corneometer units; CI, confidence interval; D0′, immediately after non‐ablative fractional laser treatment before product application; D0T10min, 10 min after product application; D0T2h, 2 h after product application; D0T30s, 30 s after product application; PU, perfusion units; SD, standard deviation.

Compared with D0′, the repair cream side showed a 4.63% reduction in TEWL at D0T30s; this beneficial change persisted through D0T2h with a 3.94% decline, alongside skin hydration increases of 89.76% and 91.86%, respectively (all *p* < 0.001) (Figure 2a,b). These parallel improvements in TEWL and hydration were greater than those on the blank control side (*p* < 0.001), whereas no significant barrier recovery was detected on the blank control side during the 2‐h observation window.

**FIGURE 2:**
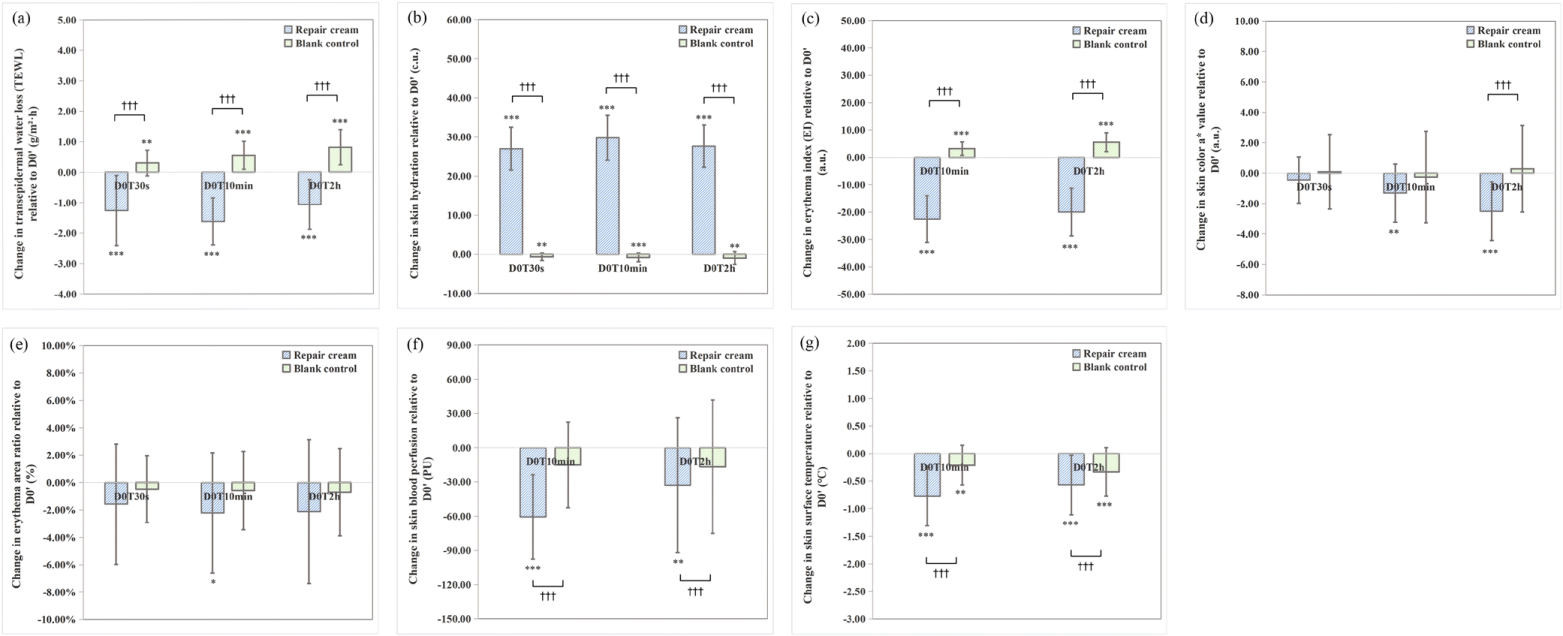
Short‐term changes of skin biophysical parameters relative to D0′. (a) Transepidermal water loss (TEWL). (b) Skin hydration. (c) Erythema index (EI). (d) Skin color a* value. (e) Erythema area ratio. (f) Skin blood perfusion. (g) Skin surface temperature. Data are expressed as mean ± SD. Statistical significance of within‐side changes versus D0′: *p < 0.05, **p < 0.01, ***p < 0.001. Between‐side *p*‐values compare repair cream versus blank control sides: ^†††^ *p* < 0.001. a.u., arbitrary units; c.u., Corneometer units; PU, perfusion units; SD, standard deviation. D0′, immediately after non‐ablative fractional laser treatment before product application; D0T30s, D0T10min, and D0T2h: 30 s, 10 min, and 2 h after product application.

For erythema‐related endpoints, the repair cream side achieved EI reductions of 5.96% at D0T10min and 5.29% at D0T2h (both *p* < 0.001) (Figure 2c). Skin color a* values decreased by 3.41% at D0T10min (*p* < 0.01) and 6.51% at D0T2h (*p* < 0.001), and the erythema area ratio declined by 5.10% at D0T10min (*p* < 0.05) (Figures 2d,e and 3). In contrast, the blank control side exhibited EI elevations of 0.85% at D0T10min and 1.47% at D0T2h (both *p* < 0.001), with no significant changes in other erythema parameters (all *p* > 0.05). Improvements in EI on the repair cream side outperformed the blank control side at D0T10min, and greater declines in EI and skin color a* value were also observed on the repair cream side at D0T2h (all *p* < 0.001).

**FIGURE 3:**
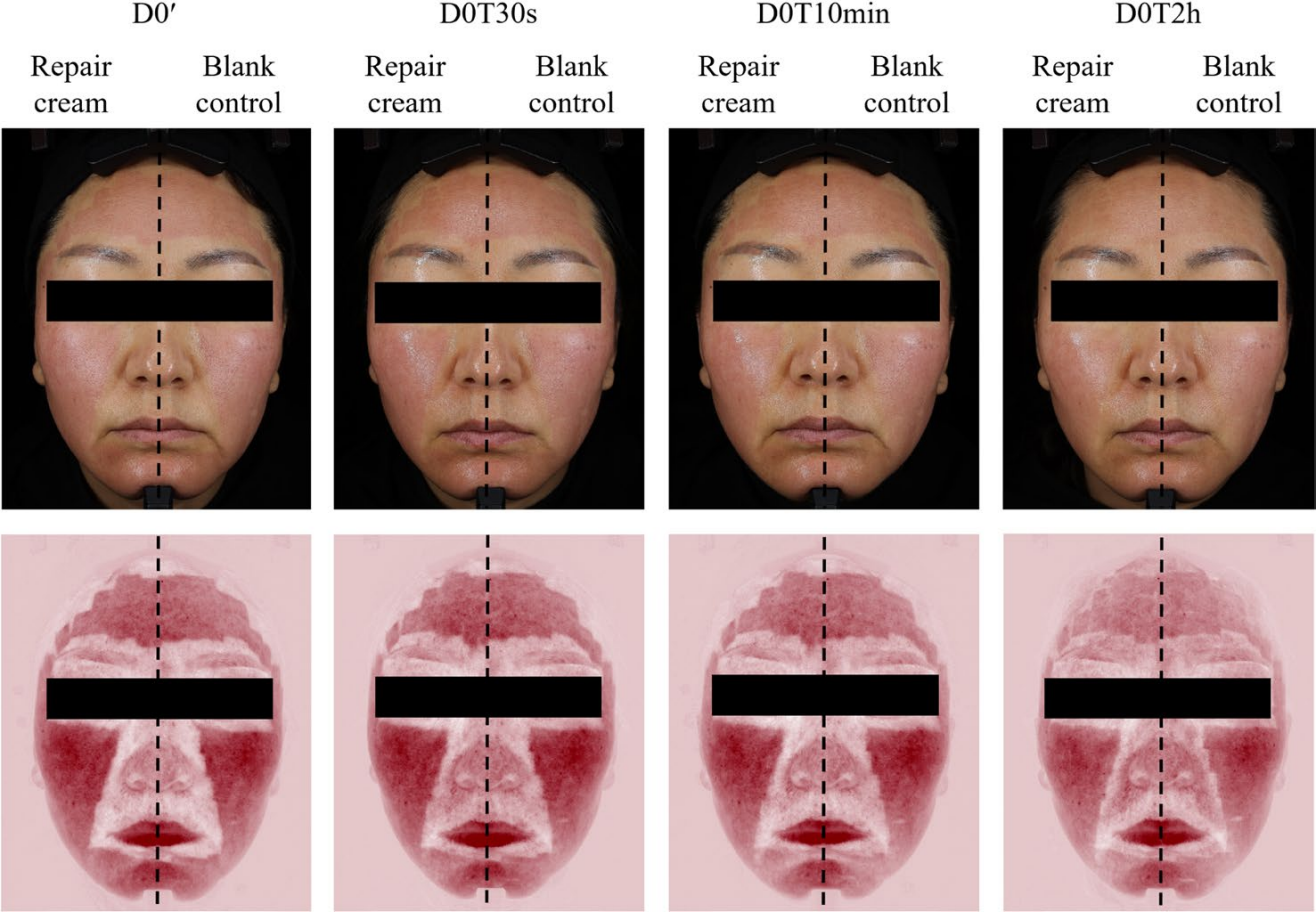
Representative short‐term erythema findings within 2 h following 1565‐nm non‐ablative fractional laser (NAFL) treatment from a split‐face trial. Standard photographs (upper row) and RBX‐mode images (lower row) were obtained using the VISIA‐CR 5.0 system. Deeper red color indicates more pronounced subcutaneous inflammation. D0′, immediately after NAFL treatment before product application; D0T30s, D0T10min, and D0T2h: 30 s, 10 min, and 2 h after product application.

Regarding cutaneous microcirculation, skin blood perfusion decreased more markedly on the repair cream side versus the blank control side at D0T10 min and D0T2h (all *p* < 0.001) (Figure 2f). Specifically, perfusion dropped by 17.85% at D0T10min (*p* < 0.001) and partially rebounded to a 9.72% reduction at D0T2h (*p* < 0.01), while the blank control side showed no significant change (*p* > 0.05). Representative images are shown in Figure 4a.

**FIGURE 4:**
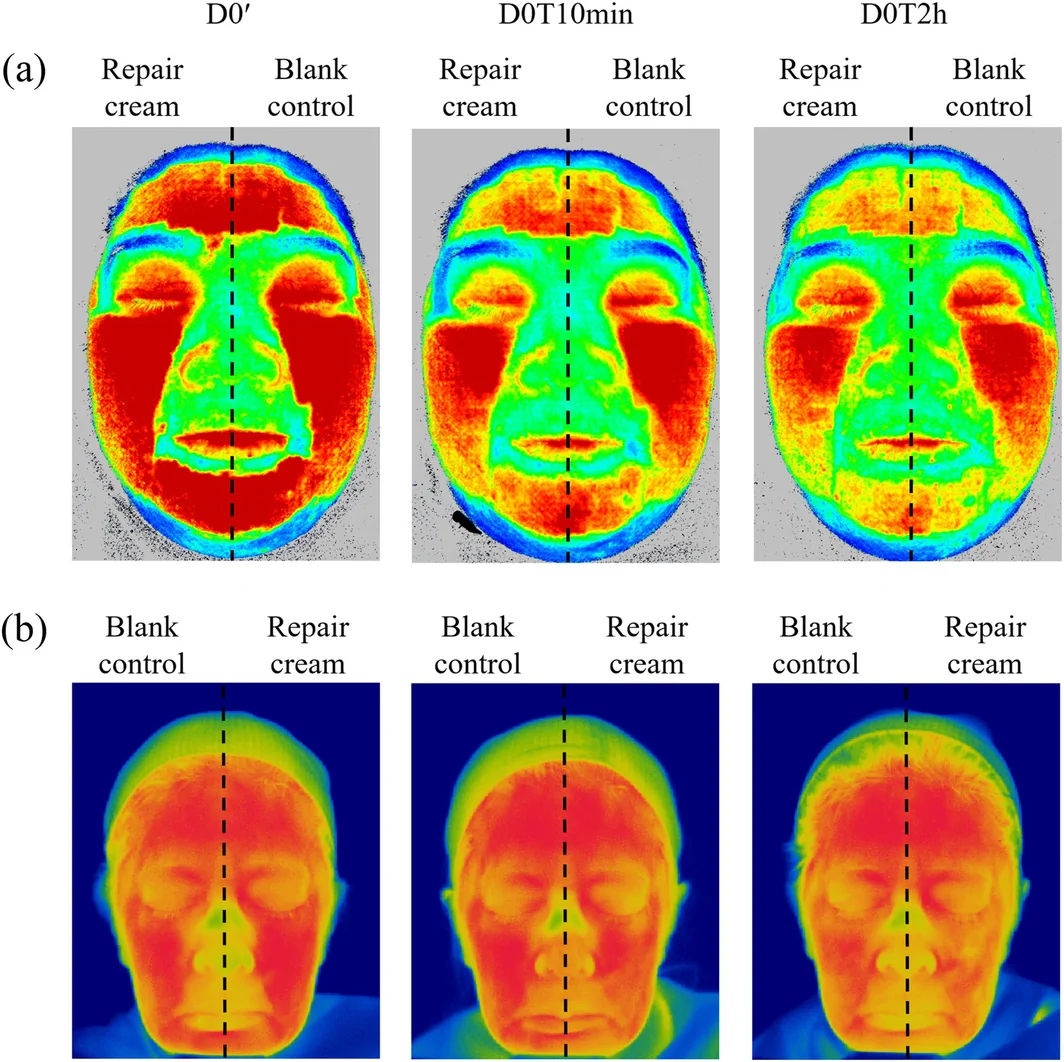
Representative short‐term skin blood perfusion and surface temperature findings within 2 h following 1565‐nm non‐ablative fractional laser (NAFL) treatment from a split‐face trial. (a) Skin blood perfusion images: Red indicates higher perfusion, and blue indicates lower perfusion. (b) Skin surface temperature maps: Brighter colors indicate higher temperature, and darker colors indicate lower temperature. D0′, immediately after NAFL treatment before product application; D0T30s, D0T10min, and D0T2h: 30 s, 10 min, and 2 h after product application.

For skin surface temperature, significant temperature reductions of 2.15% at D0T10min and 1.59% at D0T2h were observed on the repair cream side (both *p* < 0.001) (Figure 2g). The blank control side also displayed mild but significant cooling: 0.59% at D0T10min (*p* < 0.01) and 0.92% at D0T2h (*p* < 0.001), with the magnitude of temperature reduction markedly smaller than that seen on the repair cream side at both time points (both *p* < 0.001). The representative changes in surface temperature on both sides are shown in Figure 4b.

### 3.3 Medium‐Term Outcomes (D0 to D14)

Compared with D0, TEWL remained elevated on both the repair cream and standard‐care comparator sides at D3 and D7. At D14, TEWL decreased by 4.34% on the repair cream side and 1.12% on the standard‐care comparator side (both *p* < 0.001), with significantly greater barrier restoration on the repair cream side (*p* < 0.001) (Figure 5a). This temporal pattern suggests that barrier recovery to baseline levels may require approximately 14 days after 1565‐nm NAFL treatment. Concurrently, skin hydration on the repair cream side increased progressively, reaching 32.32% above baseline at D14, whereas the standard‐care comparator side showed a more modest increase of 8.51% (Table 4, Figure 5b). The hydrating effect of the repair cream was significantly superior to that of the standard‐care comparator across the 14‐day period (*p* < 0.001).

**FIGURE 5:**
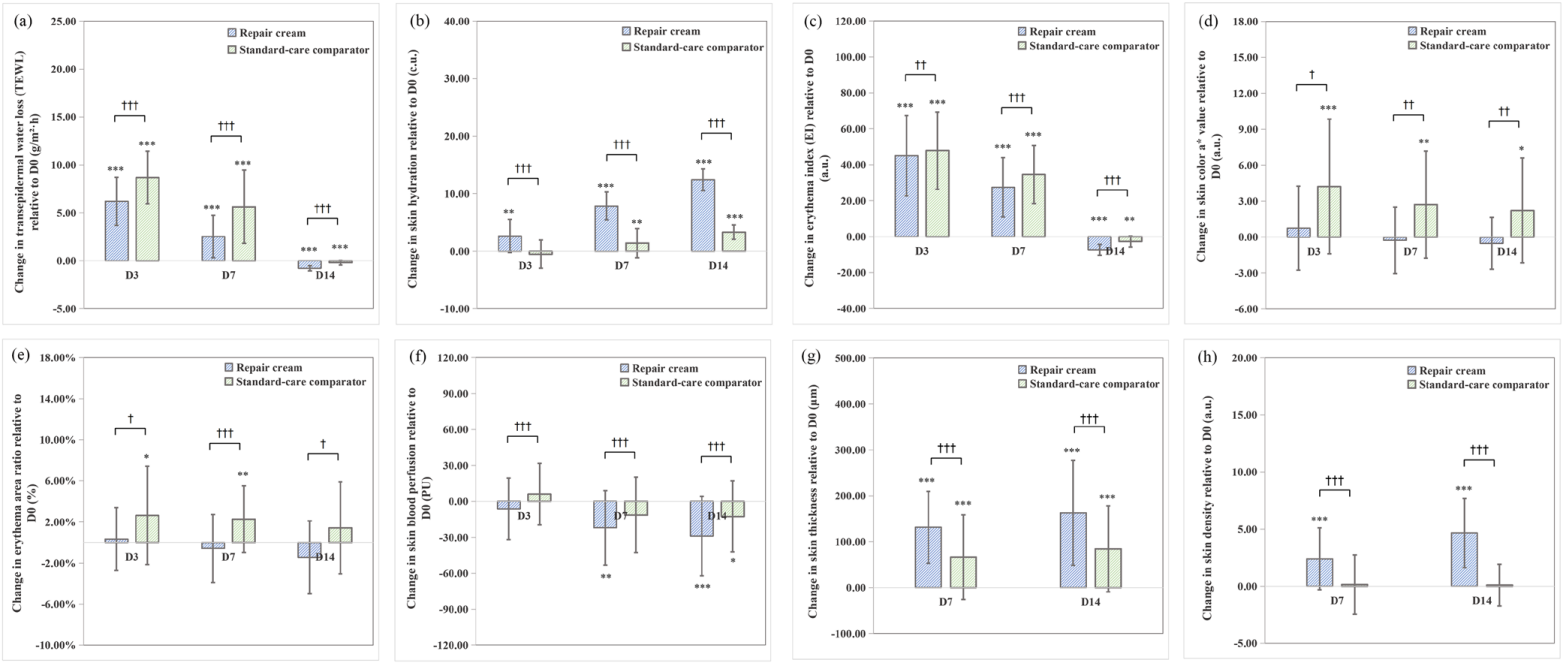
Medium‐term changes of skin biophysical parameters relative to D0. (a) Transepidermal water loss (TEWL). (b) Skin hydration. (c) Erythema index (EI). (d) Skin color a* value. (e) Erythema area ratio. (f) Skin blood perfusion. (g) Skin thickness. (h) Skin density. Data are expressed as mean ± SD. Statistical significance of within‐side changes versus D0: *p < 0.05, **p < 0.01, ***p < 0.001. Between‐side *p*‐values compare repair cream versus standard‐care comparator sides: ^†^ *p* < 0.05, ^††^ *p* < 0.01, ^†††^ *p* < 0.001. a.u., arbitrary units; c.u., Corneometer units; PU, perfusion units; SD, standard deviation.

**TABLE 4:** Medium‐term changes of skin biophysical parameters relative to D0: Repair cream versus standard‐care comparator split‐face comparison.

Parameters	Time point	Mean ± SD		Change from D0 [mean difference; 95% CI]		Between‐side *p*
		Repair cream	Standard‐care comparator	Repair cream	Standard‐care comparator	
Transepidermal water loss (TEWL) (g/m^2^·h)	D3	24.16 ± 2.70	26.60 ± 3.31	6.20 (5.31, 7.08)***	8.68 (7.71, 9.65)***	< 0.001^†††^
	D7	20.49 ± 2.55	23.55 ± 4.08	2.52 (1.74, 3.30)***	5.63 (4.27, 6.98)***	< 0.001^†††^
	D14	17.19 ± 1.44	17.72 ± 1.36	−0.78 (−0.87, −0.68)***	−0.20 (−0.29, −0.11)***	< 0.001^†††^
Skin hydration (c.u.)	D3	41.07 ± 4.28	38.27 ± 4.83	2.64 (1.62, 3.65)**	−0.52 (−1.39, 0.34)	< 0.001^†††^
	D7	46.27 ± 4.29	40.19 ± 4.96	7.84 (6.98, 8.70)***	1.40 (0.50, 2.29)**	< 0.001^†††^
	D14	50.86 ± 3.58	42.09 ± 3.90	12.42 (11.76, 13.09)***	3.30 (2.85, 3.75)***	< 0.001^†††^
Erythema index (EI) (a.u.)	D3	376.48 ± 43.66	379.12 ± 43.19	45.09 (37.15, 53.03)***	47.86 (40.23, 55.48)***	0.003^††^
	D7	358.84 ± 41.62	365.78 ± 41.83	27.45 (21.58, 33.33)***	34.52 (28.79, 40.24)***	< 0.001^†††^
	D14	324.05 ± 44.77	328.56 ± 43.67	−7.33 (−8.41, −6.25)***	−2.71 (−3.79, −1.62)**	< 0.001^†††^
Skin color a* value (a.u.)	D3	23.37 ± 6.36	27.23 ± 6.92	0.74 (−0.51, 1.98)	4.22 (2.23, 6.22)***	0.010^†^
	D7	22.37 ± 5.91	25.73 ± 6.51	−0.27 (−1.25, 0.72)	2.72 (1.14, 4.30)**	0.001^††^
	D14	22.10 ± 4.73	25.23 ± 5.99	−0.53 (−1.30, 0.23)	2.22 (0.66, 3.77)*	0.001^††^
Erythema area ratio (%)	D3	40.61 ± 4.72	41.70 ± 6.54	0.33 (−0.75, 1.42)	2.64 (0.94, 4.34)*	0.045^†^
	D7	39.55 ± 4.80	41.30 ± 4.86	−0.58 (−1.75, 0.60)	2.27 (1.12, 3.42)**	< 0.001^†††^
	D14	38.91 ± 3.79	40.52 ± 6.11	−1.45 (−2.71, −0.19)	1.42 (−0.16, 3.01)	0.030^†^
Skin blood perfusion (PU)	D3	129.22 ± 25.54	142.20 ± 32.51	−6.25 (−15.32, 2.81)	6.10 (−2.99, 15.18)	< 0.001^†††^
	D7	113.46 ± 21.63	124.74 ± 27.59	−22.01 (−33.03, −10.99)**	−11.37 (−22.52, −0.22)	< 0.001^†††^
	D14	106.59 ± 18.74	123.54 ± 21.42	−28.88 (−40.65, −17.12)***	−12.56 (−23.08, −2.05)*	< 0.001^†††^
Skin thickness (μm)	D7	1416.36 ± 171.56	1336.27 ± 169.87	131.21 (103.44, 158.98)***	66.21 (33.48, 98.95)***	< 0.001^†††^
	D14	1447.94 ± 184.66	1354.15 ± 172.12	162.79 (122.30, 203.28)***	84.09 (50.93, 117.25)***	< 0.001^†††^
Skin density (a.u.)	D7	28.72 ± 7.77	26.63 ± 8.25	2.40 (1.44, 3.35)***	0.15 (−0.77, 1.07)	< 0.001^†††^
	D14	30.98 ± 7.41	26.52 ± 7.57	4.66 (3.59, 5.73)***	0.04 (−0.61, 0.69)	< 0.001^†††^

*Note:* Statistical significance of within‐side changes versus D0: *p < 0.05, **p < 0.01, ***p < 0.001. Between‐side *p*‐values compare repair cream versus standard‐care comparator sides: ^†^ *p* < 0.05, ^††^ *p* < 0.01, ^†††^ *p* < 0.001.

Abbreviations: a.u., arbitrary units; c.u., Corneometer units; CI, confidence interval; PU, perfusion units; SD, standard deviation.

Consistent with TEWL kinetics, EI increased on both sides at D3 and D7 before declining by D14. At D14, EI decreased by 2.21% on the repair cream side (*p* < 0.001) and by 0.82% on the standard‐care comparator side (*p* < 0.01), with significantly greater improvement on the repair cream side (*p* < 0.001) (Figure 5c). Skin color a* value and erythema area ratio followed divergent patterns between the two sides. On the repair cream side, both parameters trended downward from D7 to D14, though neither change reached statistical significance. In contrast, the standard‐care comparator side exhibited persistent elevation of both parameters through D14 without return to baseline, yielding significantly better anti‐erythema outcomes on the repair cream side (*p* < 0.001) (Figures 5d,e and 6a).

**FIGURE 6:**
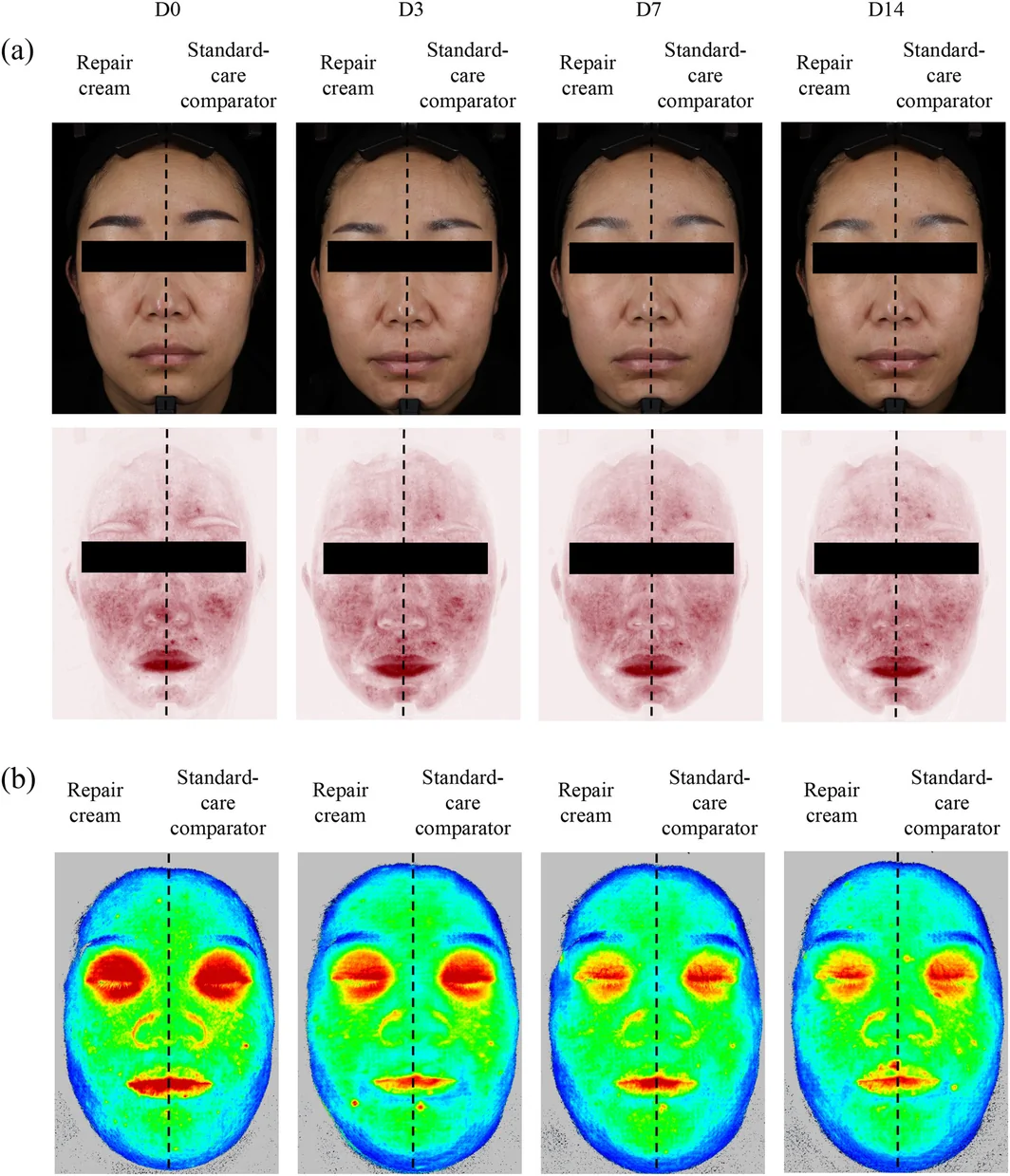
Representative medium‐term erythema and skin blood perfusion findings following 1565‐nm non‐ablative fractional laser treatment from a split‐face trial. (a) Standard photographs (upper row) and RBX‐mode images (lower row); deeper red indicates more pronounced subcutaneous inflammation. (b) Skin blood perfusion maps: red indicates higher perfusion, and blue indicates lower perfusion. Assessments were conducted at baseline (D0) and on days 3, 7, and 14 (D3, D7, D14) post‐treatment.

For cutaneous microcirculation, skin blood perfusion on the repair cream side decreased by 16.25% at D7 (*p* < 0.01), and 21.32% at D14 (*p* < 0.001) compared with D0 (Figures 5f and 6b). Significant perfusion reductions were observed on the standard‐care comparator side only at D14 (9.23%, *p* < 0.05). Perfusion decline was substantially more pronounced on the repair cream side at D7 and D14 (*p* < 0.001).

Ultrasonographic dermal parameters showed greater change on the repair cream side. Relative to D0, skin thickness increased by 10.21% at D7 and 12.67% at D14, while skin density rose by 9.12% and 17.70% at D7 and D14, respectively (all *p* < 0.001) (Figure 5g,h). The standard‐care comparator side also showed significant thickness increases of 5.21% and 6.62% at D7 and D14 (both *p* < 0.001), but no significant change in skin density (*p* > 0.05). Representative ultrasound images are shown in Figure 7. Collectively, ultrasonographic dermal parameters showed greater change on the repair cream side (*p* < 0.001).

**FIGURE 7:**
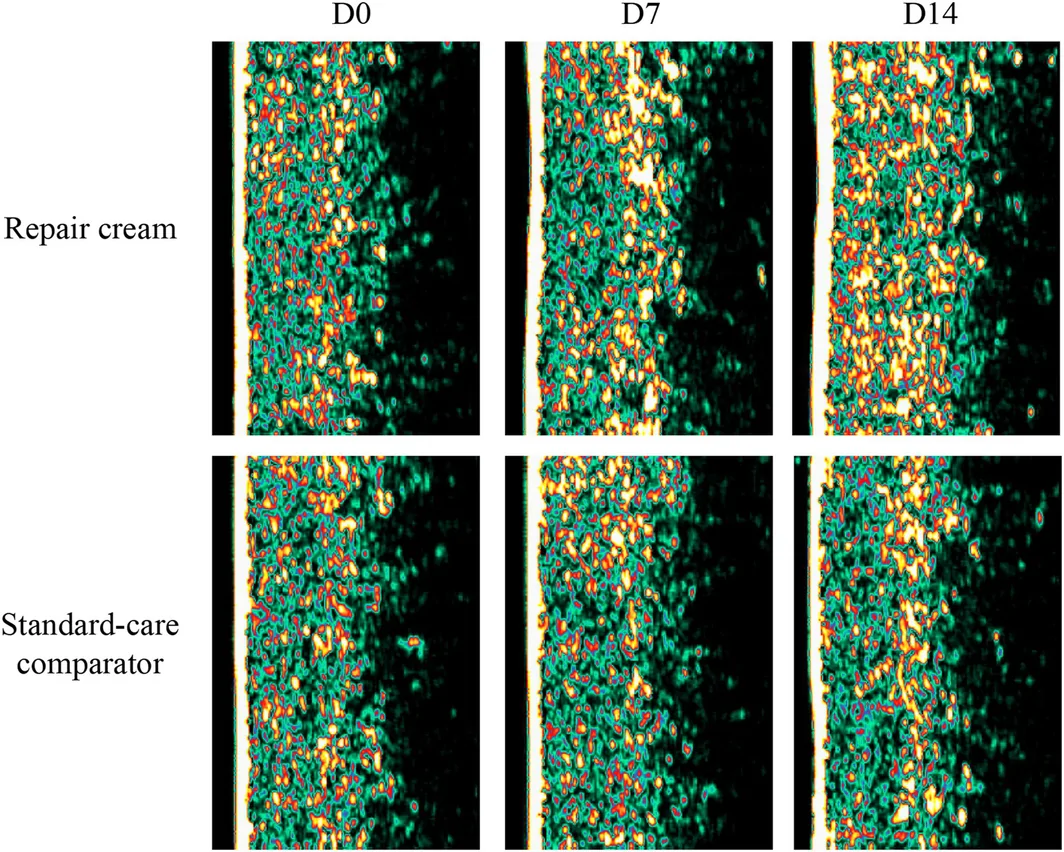
Representative medium‐term dermal structural findings following 1565‐nm non‐ablative fractional laser treatment from a split‐face trial. Increased yellow or bright fluorescence intensity reflects higher protein content, and a larger fluorescent area indicates a stronger collagen response. Assessments were conducted at baseline (D0) and on days 7 and 14 (D7, D14) post‐treatment.

### 3.4 Safety Assessment

No product‐related adverse events or serious adverse events were observed by the dermatologist investigators or reported by participants throughout the 14‐day study. All participants reported no discomfort, irritation, or other symptoms attributed to the repair cream, and all participants rated the product as mild and non‐irritating. These findings collectively indicate that the repair cream was well tolerated and demonstrated a favorable safety profile in this post‐NAFL setting.

## 4 Discussion

This prospective, randomized, investigator‐blind, split‐face controlled trial provides the first comprehensive evidence that a repair cream containing dexpanthenol, rhFNP, and *Coreopsis tinctoria* extract delivers dual‐phase therapeutic value following 1565‐nm NAFL treatment. Specifically, it facilitates immediate ultra‐early inflammatory modulation within the critical 0–2 h post‐procedural window and induces favorable early changes in ultrasonographic dermal parameters over 14 days. The study design, which incorporates a blank control for short‐term assessment and a standard‐of‐care comparator for medium‐term evaluation, establishes a robust evidence framework for integrating this formulation into post‐NAFL management protocols.

The immediate 0–2 h window following 1565‐nm NAFL treatment is a critical therapeutic interval. Thermal damage induced by microscopic thermal zones initiates oxidative stress, inflammatory cytokine release, and capillary dilation, routinely triggering clinical erythema, edema, and acute barrier disruption across treated skin [6, 12, 13, 16, 34, 35]. On the blank control side, the absence of active intervention allowed this expected pathophysiology to persist, with EI and TEWL remaining elevated throughout the 0–2 h window. This confirms that without timely management, the inflammatory cascade progresses unchecked during this critical period, as posited in the Introduction. In contrast, the repair cream rapidly lowered skin surface temperature by 2.15% and skin blood perfusion by 17.85% at 10 min, while boosting hydration by 89.76% at 30 s and reducing TEWL. These immediate improvements reflect dual mechanisms: physical occlusive moisture retention and targeted anti‐inflammatory signaling. Dexpanthenol penetrates the stratum corneum, converts to pantothenic acid to restore lipid matrix integrity and reduce TEWL [26, 36], and concurrently upregulates heme oxygenase‐1 and matrix metalloproteinase 3 (MMP3) to attenuate reactive oxygen species‐mediated inflammatory signaling after thermal injury [37]. rhFNP, through arginine‐glycine‐aspartic acid (RGD)‐mediated integrin engagement, stabilizes the provisional ECM and suppresses the release of pro‐inflammatory cytokines, including tumor necrosis factor‐α, interleukin‐1β, and interleukin‐6 [27, 28, 38] Okanin, the major active component of *Coreopsis tinctoria* , inhibits the toll‐like receptor 4/nuclear factor κB signaling cascade to reduce pro‐inflammatory cytokine secretion and preserve mitochondrial integrity [32, 33], while the total extract of *Coreopsis tinctoria* exerts antioxidative effects by activating the nuclear factor erythroid 2‐related factor 2 pathway [30]. Collectively, these bioactive components attenuate the acute inflammatory response, as reflected in the concurrent reduction of EI, skin surface temperature, blood perfusion, and TEWL within the 2‐h post‐application window.

The findings confirm the rapid regulatory effects of the test formulation on epidermal barrier function and microcirculation during the post‐laser golden window. We further employed 20‐MHz ultrasound to evaluate medium‐term dermal changes, establishing a multi‐dimensional assessment system that encompasses both epidermal function and ultrasonographic dermal assessment. As noted above, this design addresses a recognized limitation in the existing literature: the scarcity of quantitative dermal endpoints in post‐laser skincare trials. On day 14, the treatment side exhibited significant increases in dermal thickness (12.67%) and density (17.70%). In contrast, the blank control side only demonstrated a mild elevation in dermal thickness (6.62%) and showed no statistically significant changes in dermal density. This intergroup difference indicates that the bioactive ingredients are associated with ultrasonographic changes not attributable to passive hydration alone.

Conventional postoperative care utilizing hyaluronic acid dressings and bland moisturizers merely provides physical occlusive protection without bioactive regulation of dermal matrix synthesis [13]. Mechanistically, rhFNP upregulates the expression of Type I and III collagen while inhibiting MMP activity, shifting the balance toward ECM accumulation rather than degradation [27, 38, 39]. Dexpanthenol stimulates fibroblast proliferation and granulation tissue formation, further supporting sustained dermal repair and remodeling [23, 36]. That said, the two ultrasonographic parameters warrant distinct interpretation. The sharp increase in dermal thickness immediately after NAFL (D0′: approximately +28% versus baseline) predominantly reflects acute thermal edema rather than new matrix accretion; consequently, the day 14 value represents a point along the edema resolution curve, and thickness alone is an unreliable indicator of structural remodeling at this early time point. Dermal density behaves differently. Edema reduces echogenicity, as confirmed by the significant bilateral density decrease at D0′. The restoration of density on the treatment side to +17.70% above baseline at D14, in contrast to no significant change on the control side, cannot be attributed to residual edema. We therefore regard density, rather than thickness, as the more informative parameter for detecting early matrix‐level effects in this timeframe. It should be noted, however, that high‐frequency ultrasound quantifies acoustic impedance rather than collagen subtype or fiber organization [40, 41], and the complete remodeling trajectory extends beyond the current 14‐day window, as discussed below.

In this study, we quantified a sequential pathological progression following 1565‐nm NAFL irradiation, including thermal damage, inflammatory activation, skin barrier disruption, microcirculatory hyperactivity, and subsequent dermal structural regeneration. This unified assessment protocol allows for the simultaneous monitoring of epidermal barrier function, dermal matrix architecture, skin blood perfusion, and surface temperature.

As highlighted in the introduction, individuals with sensitive skin exhibit heightened neurovascular responses and possess inherently fragile barriers, making them more susceptible to adverse reactions following laser treatment. Unfortunately, this population is often underrepresented in relevant quantitative and controlled studies. To address this research gap, we strictly enrolled participants with baseline skin barrier impairment (TEWL > 15 g/m^2^·h, corneometer < 45 c.u., facial erythema grade ≥ 3 per standardized photographic atlas, self‐reported sensitive facial skin). This enrollment criterion established a rigorous stress model to simulate real‐world scenarios of sensitive skin undergoing NAFL treatment. The combination of pre‐existing barrier dysfunction and laser‐induced thermal injury created a challenging recovery environment. Nevertheless, the test repair cream demonstrated immediate soothing effects and sustained structural improvements in this vulnerable population without causing adverse events, providing robust clinical evidence for its favorable tolerability and efficacy. Our findings address two critical clinical concerns: whether patients with sensitive skin can safely undergo NAFL treatment and what optimized post‐procedural regimen can enhance therapeutic outcomes. Collectively, the data confirm that with appropriate topical intervention, skin with pre‐existing barrier impairment can achieve favorable recovery and exhibit early dermal ultrasonographic changes following NAFL treatment.

The control regimen consisted of a medical sodium hyaluronate dressing applied for three consecutive days followed by a basic moisturizing cream—a standard post‐NAFL care pathway widely used in clinical practice. Compared to this stepped, multi‐product protocol, the repair cream achieved superior outcomes across all detected indices while requiring only twice‐daily application of a single product over 14 days. As an ancillary benefit, this simplification of postoperative care may improve patient adherence and reduce the cumulative cost of multiple products. However, the primary clinical value of this formulation lies in its active, multi‐targeted modulation of both acute inflammation and dermal ultrasonographic parameters, rather than in regimen convenience alone. This is particularly relevant for sensitive‐skin patients, given the demonstrated efficacy and safety in a compromised barrier setting.

Several limitations of this study should be acknowledged when interpreting the current results. First, the single‐center design and relatively modest sample size (*n* = 33) may restrict the generalizability of the findings to broader populations. Second, all enrolled participants were female, so the results cannot be directly extrapolated to male patients, who may present distinct inflammatory responses and skin healing kinetics. Third, the 14‐day follow‐up period captures only the earliest phase of dermal remodeling; as noted in the preceding discussion, high‐frequency ultrasound quantifies acoustic impedance rather than collagen subtype or fiber organization [40, 41], and histological studies have established that neocollagenesis and matrix reorganization continue for at least three months after fractional photothermolysis [8, 16, 34, 42]. Fourth, while this study prioritized non‐invasive in vivo assessments, the absence of tissue sampling precludes direct verification of cellular and molecular mechanisms—particularly intracellular oxidative stress dynamics, inflammatory cytokine kinetics, and the transcriptional pathways activated by rhFNP and dexpanthenol in the post‐laser wound environment. Although the split‐face design effectively controls for inter‐individual variability, systemic inflammatory mediators released following laser irradiation may still exert bilateral influences that cannot be fully discriminated by the current design. Future research should conduct multi‐center randomized controlled trials with larger cohorts including both male and female participants, as well as extended follow‐up to at least 3 months to capture the complete dermal remodeling trajectory. Mechanistic studies combining serial skin biopsies with transcriptomic and proteomic analyses are needed to clarify the molecular pathways underlying the observed ultrasonographic improvements. Furthermore, head‐to‐head comparative trials with other bioactive post‐laser formulations will help further define the clinical positioning of this combined topical product within laser adjunctive care.

## 5 Conclusions

This prospective, randomized, investigator‐blind, split‐face controlled trial demonstrates that a repair cream containing dexpanthenol, rhFNP, and *Coreopsis tinctoria* extract delivers dual‐phase therapeutic value following 1565‐nm NAFL treatment. The formulation mitigates acute inflammatory responses during the 0–2 h post‐procedural window, as evidenced by reductions in skin surface temperature, skin blood perfusion, EI, and TEWL, alongside an increase in hydration levels. Over 14 days of continued use, it is associated with significantly greater recovery of epidermal barrier function and with a favorable early divergence in ultrasonographically assessed dermal parameters—most notably a recovery of dermal density to above baseline that was not observed with the comparator regimen, an early signal warranting longer follow‐up and histological confirmation. The product was well tolerated, with no adverse events reported in participants with pre‐existing barrier compromise, supporting its use in sensitive skin populations.

## Author Contributions

Conceptualization: R.H. and Z.Y. Methodology: R.H. and X.L. Formal analysis: M. Lu. Resources: R.H., Z.Y. and M. Li. Data curation: X.L. and M. Lu. Writing – original draft preparation: Q.L. Writing – review and editing: R.H. and P.S. Visualization: M. Lu. Supervision: R.H., P.S. and Z.Y. All authors have read and agreed to the published version of the manuscript.

## Ethics Statement

The study protocol, informed consent form, and case report forms were approved by the Ethics Committee of the Medical Laboratory of SGS Standards and Technology (Qingdao) Co. Ltd. (approval no: QDMED2025114). Before participating in any study‐related procedures, all participants provided a written informed consent form following full disclosure of the study objectives, methodologies, potential risks, and their right to withdraw from the study at any time without repercussions.

## Conflicts of Interest

The authors declare no conflicts of interest.

## Data Availability

The data that support the findings of this study are available from the corresponding author upon reasonable request.
